# Risk of low bone mineral density and low body mass index in patients with non-celiac wheat-sensitivity: a prospective observation study

**DOI:** 10.1186/s12916-014-0230-2

**Published:** 2014-11-28

**Authors:** Antonio Carroccio, Maurizio Soresi, Alberto D’Alcamo, Carmelo Sciumè, Giuseppe Iacono, Girolamo Geraci, Ignazio Brusca, Aurelio Seidita, Floriana Adragna, Miriam Carta, Pasquale Mansueto

**Affiliations:** Department of Internal Medicine, University Hospital of Palermo, Via del Vespro 141, Palermo, Italy; Department of Internal Medicine, Giovanni Paolo II Hospital, Via Pompei, Sciacca, Italy; Department of Surgery, Oncology, and Odontology, University of Palermo, Via L. Giuffrè 5, Palermo, Italy; Pediatric Gastroenterology, Di Cristina Hospital Palermo, Piazza P. Montalto 2, Palermo, Italy; Department of Pathology, Buccheri - La Ferla Hospital Palermo, Via Messina Marine 197, Palermo, Italy

**Keywords:** Non-celiac wheat sensitivity, Multiple food allergy, Body mass index, Osteoporosis

## Abstract

**Background:**

Non-celiac gluten sensitivity (NCGS) or ‘wheat sensitivity’ (NCWS) is included in the spectrum of gluten-related disorders. No data are available on the prevalence of low bone mass density (BMD) in NCWS. Our study aims to evaluate the prevalence of low BMD in NCWS patients and search for correlations with other clinical characteristics.

**Methods:**

This prospective observation study included 75 NCWS patients (63 women; median age 36 years) with irritable bowel syndrome (IBS)-like symptoms, 65 IBS and 50 celiac controls. Patients were recruited at two Internal Medicine Departments. Elimination diet and double-blind placebo controlled (DBPC) wheat challenge proved the NCWS diagnosis. All subjects underwent BMD assessment by Dual Energy X-Ray Absorptiometry (DXA), duodenal histology, HLA DQ typing, body mass index (BMI) evaluation and assessment for daily calcium intake.

**Results:**

DBPC cow's milk proteins challenge showed that 30 of the 75 NCWS patients suffered from multiple food sensitivity. Osteopenia and osteoporosis frequency increased from IBS to NCWS and to celiac disease (CD) (*P* <0.0001). Thirty-five NCWS patients (46.6%) showed osteopenia or osteoporosis. Low BMD was related to low BMI and multiple food sensitivity. Values of daily dietary calcium intake in NCWS patients were significantly lower than in IBS controls.

**Conclusions:**

An elevated frequency of bone mass loss in NCWS patients was found; this was related to low BMI and was more frequent in patients with NCWS associated with other food sensitivity. A low daily intake of dietary calcium was observed in patients with NCWS.

**Electronic supplementary material:**

The online version of this article (doi:10.1186/s12916-014-0230-2) contains supplementary material, which is available to authorized users.

## Background

Celiac disease (CD) has been reported to increase the risk of osteoporosis, with a resulting augmented risk of fractures [1]. More recently, it has been reported that a consistent percentage of the general population consider themselves to be suffering from problems caused by wheat and/or gluten ingestion, even though they do not have CD or wheat allergy [2,3]. This clinical condition has been named Non-Celiac Gluten Sensitivity’ (NCGS). In a previous paper [4] we suggested the term ‘Non-Celiac Wheat Sensitivity’ (NCWS), since it is not known what component of wheat causes the symptoms in NCGS patients, and we also showed that these patients had a high frequency of coexistent multiple food hypersensitivity [4]. That previous study also showed a percentage of NCWS patients had weight loss and anemia: whether these depended on the intestinal malabsorption or not remains unclear.

As yet no data are available on the presence and prevalence of low bone mass density (BMD) in NCWS patients. The aims of the present study were: 1) to investigate the prevalence of low BMD in NCWS patients; and 2) to search for a possible correlation between BMD and other clinical characteristics, in particular the body mass index (BMI), of NCWS patients.

## Methods

### Study population

This prospective study included consecutive adult patients with an irritable bowel syndrome (IBS)-like clinical presentation, according to Rome II criteria [5], and a definitive diagnosis of NCWS. The patients were recruited at the Internal Medicine Department of the University Hospital of Palermo and at the Internal Medicine Department of the Hospital of Sciacca (Italy), between July 2011 and July 2013. Most of the patients were referred due to intestinal symptoms with onset after wheat ingestion. None of them were included in previous published studies.

NCWS diagnosis was made according to the recently proposed criteria [2] (see Additional file 1). Both the CD specific serum antibodies and the duodenal histology were evaluated on a diet containing at least 100 grams of wheat per day, for at least three months. Furthermore, according to our experience [4], all patients met the following adjunctive inclusion criteria: 1) resolution of the IBS symptoms on a standard elimination diet excluding wheat, cow’s milk, egg, tomato, chocolate and other self-reported food(s) causing symptoms; and 2) symptom reappearance on double-blind placebo-controlled (DBPC) wheat challenge. DBPC cow’s milk protein (CMP) challenge and other ‘open’ food challenges were also performed in all cases [4].

Exclusion criteria were: 1) self-exclusion of wheat from the diet and refusal to reintroduce it, before entering the study; 2) nervous system disease, major psychiatric disorder; 3) hyperparathyroidism, Cushing’s syndrome, kidney disease and other ‘organic’ gastrointestinal disorders; 4) physical impairment limiting physical activity; 5) menopause; and 6) steroid and sex steroid therapy, hormone replacement therapy or ovariectomy.

Fifty sex- and age-matched subjects with CD and sixty-five suffering from IBS unrelated to NCWS or other food ‘intolerance,’ diagnosed according to standard criteria during the same study period, were chosen at random (randomized by computer generated method among the patients with a new CD or IBS diagnosis posed during the same study period) and enrolled as control groups. The same exclusion criteria used for the NCWS patients were applied to the control groups.

### Methods

On entering the study all patients underwent serum assays and HLA-DQ typing for CD diagnosis (see Additional file 1). Specific IgE (RAST) and/or skin prick tests for food allergens were performed on all patients, as previously described [4].

### Duodenal histology

Four to six biopsy specimens were obtained from the bulb and the second duodenal portion during gastroduodenoscopy, performed when the patients were on a wheat-containing diet. The slides were stained with hematoxylin and eosin and graded according to the original modified Marsh classification [6]. The number of intraepithelial lymphocytes (IELs) per 100 villous epithelial cells was assessed by immunohistochemical staining. The upper limit of the reference interval in our laboratory was 25 IEL/100 epithelial cells. The number of eosinophils (EOS) per high-power fields (HPFs, 40 X) was also assessed; the upper limit of the reference interval in our laboratory was 60 EOS/10 HPFs.

### Elimination diet and double-blind placebo-controlled challenge method

On entering the study all patients commenced a standard elimination diet, with the exclusion of wheat, cow’s milk, eggs, tomato and chocolate [4]. Patients self-reporting food hypersensitivity were also asked to avoid ingestion and/or contact with the food(s) causing symptoms. After four weeks on the elimination diet they underwent DBPC wheat challenges, as previously described [4] (see Additional file 1). During all phases of the study, including the challenge period, the severity of symptoms was recorded: the patients completed a 100 mm visual analog scale (VAS), with 0 representing no symptoms, which assessed overall symptoms and the specific symptoms they each reported. DBPC challenge for CMPs was performed using an identical method [4], at least four weeks before or after wheat challenge and when the VAS score was <10, on the elimination diet. The challenges were stopped when clinical reactions occurred (increase in VAS score >30) for at least two consecutive days (onset of abdominal discomfort or pain, associated with a change in stool frequency and/or appearance). The challenges were considered positive if the same symptoms which had been initially present reappeared after their disappearance on the elimination diet.

### Other laboratory examinations

The nutritional status of each patient was defined at diagnosis by measuring the following laboratory indices: hemoglobin, serum albumin and triglycerides.

### Bone mineral density assessment

BMD was assessed at baseline by Dual Energy X-Ray Absorptiometry (DXA), using a QDR Discovery Hologic DXA in the femoral neck and in the lumbar spine by total body DXA. For each scan, BMD (expressed as absolute values in g/cm^2^) and T-scores were recorded (for details see Additional file 1). Repeated measurements of the lumbar spine of ten healthy volunteers were performed to evaluate the *in vivo* precision of the absorptiometer. The average coefficient of variation was 2.1%.

### Physical examination and dietary assessment

At the start of the study, the BMI of all participants was recorded. The subjects also filled in a health and lifestyle questionnaire considering menarche, medical history and lifestyle habits (including physical activity, smoking and elimination diet, that is, wheat and other foods). The subjects also received a previously validated dietary form containing a printed list of the most common foods [7] (for details see Additional file 1). Final forms were analyzed using a computerized database and calcium intake was calculated using a nutrient composition database [8].

### Statistical analysis

Data were expressed as mean ± standard deviation (SD) when the distribution was Gaussian and differences were calculated using Student’s *t*-test. Otherwise, data were expressed as median and range, and analyzed with the Kruskall Wallis test and with the Mann–Whitney *U* test. Fisher’s exact and Mantel-Haenszel tests, Pearson’s correlation and Spearman's rank correlation were used where appropriate. An analysis of variance (ANOVA) test was performed to evaluate the BMD differences between the three study groups; *post hoc* analysis and comparison between two groups was performed by means of the Bonferroni test. Multiple regression analysis was performed to evaluate the association between the presence of osteopenia and osteoporosis with the other clinical variables evaluated. *P* <0.05 was considered significant. All analyses were performed using the SPSS software package (version 16.0; Chicago, IL, USA).

The study was approved by the local Ethics Committee of the University Hospital of Palermo, and informed consent was obtained from all subjects.

## Results

During the study period, 80 patients fulfilled the inclusion criteria, but 5 of them refused to undergo DXA and were excluded (see Additional file 2). The 75 patients included showed IBS-like symptoms on entering the study, which disappeared completely on the elimination diet and reappeared on DBPC wheat-challenge (VAS score increase >30) (see Additional file 3). Table 1 provides the clinical characteristics of the NCWS patient group, compared to the control groups. NCWS patients had higher frequencies of weight loss, anemia (90% had iron deficiency anemia), coexistent atopic diseases and family history of CD than IBS controls.

**Table 1:** **Clinical characteristics of the NCWS patient group, compared to the control group composed of patients with IBS unrelated to NCWS and of CD patients**

**Variables**	**NCWS Patients**	**IBS Patients**	**CD Patients**	***P*** **value**
	**(Number = 75)**	**(Number = 65)**	**(Number = 50)**	
Sex	12 M, 63 F	10 M, 55 F	8 M, 42 F	n.s.
Age range (median)	20 to 49 years (36 years)	19 to 50 years (35 years)	20 to 50 years (35 years)	n.s.
IBS type				
Diarrhea	45 (60%)	36 (55%)	20 ‘typical’	
Constipation	7 (9%)	7 (11%)	30 ‘atypical’	n.s.
Alternate bowel movements	23 (31%)	22 (34%)		
Symptom duration median (range)	5 years (1 to 30 years)	4 years (0.5 to 20 years)	4 years (0.5 to 10 years)	n.s.
Weight loss	24/75 (29%)	3/65 (4%)	15/50 (30%)	NCGS vs. IBS 0.0003
				CD vs. IBS 0.0001
				CD vs. NCGS n.s.
Anemia (Hb <12 g/dl)	12/75 (16%)	4/65 (6%)	25/50 (50%)	NCGS vs. IBS 0.02
				CD vs. IBS 0.0001
				CD vs. NCGS 0.01
Family history of CD	18/75 (24%)	0/65 (0%)	10/50 (20%)	NCGS vs. IBS 0.0002
				CD vs. IBS 0.001
				CD vs. NCGS n.s.
Coexistent atopy	23/75 (30%)	6/65 (9.5%)	10/50 (20%)	NCGS vs. IBS 0.01
				CD vs. IBS n.s.
				CD vs. NCGS n.s.

Weight loss was considered when the patients reported a loss >10% of body weight during the previous year. Family history of CD indicates a CD diagnosis in a first-degree relative. Atopic diseases were: rhinitis, conjunctivitis, bronchial asthma, atopic dermatitis. CD, celiac disease; F, female; Hb, hemoglobin; IBS, irritable bowel syndrome; M, male; NCGS, non-celiac gluten sensitivity; NCWS, non-celiac wheat sensitivity; n.s., not significant; vs., versus.

According to the results of the DBPC challenge with CMP, 30 of the 75 NCWS patients were found to have multiple food sensitivity (NCWS plus CMP sensitivity). These 30 patients were asymptomatic on the elimination diet and experienced IBS symptoms again on CMP challenge. Moreover, eight of these thirty patients experienced IBS-like symptoms after open challenges with egg (four cases), tomato (three cases) or chocolate (two cases).

HLA DQ2 or DQ8 haplotypes were present in 31 of 75 (41%) NCWS patients. The duodenal histology showed normal villi but a high number of IEL in the mucosa (Marsh 1) in 39 NCWS patients (52%), whereas the remaining 36 NCWS patients had completely normal mucosa (Marsh 0); an eosinophil infiltrate was observed in 24 of the 75 (32%) NCWS patients.

**Figure 1:**
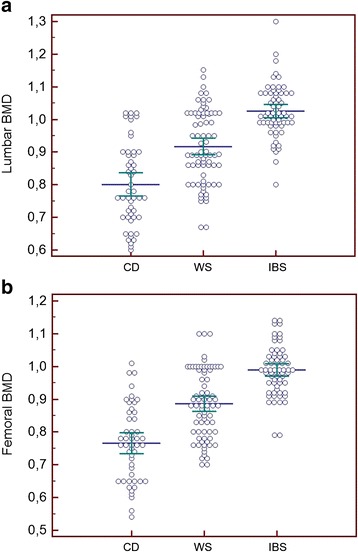
**Individual and mean ±95% c.i. (bars) values of lumbar (a) and femoral (b) BMD in the three study groups, expressed in absolute values (g/cm** ^**2**^ **).** BMD, bone mass density; c.i., confidence interval.

Figure 1 shows the individual values of BMD in the three study groups. NCWS patients had a significantly lower BMD than IBS controls both at the lumbar spine and at the femoral neck (*P* <0.0001). The patients suffering from CD showed a significantly lower BMD than NCWS patients and IBS controls both at the lumbar spine (ANOVA: F = 8.7; *P* <0.0001; Bonferroni *P* <0.0001) and at the femoral neck (ANOVA: F = 6.7; *P* <0.002; Bonferroni: *P* <0.0001).

**Figure 2:**
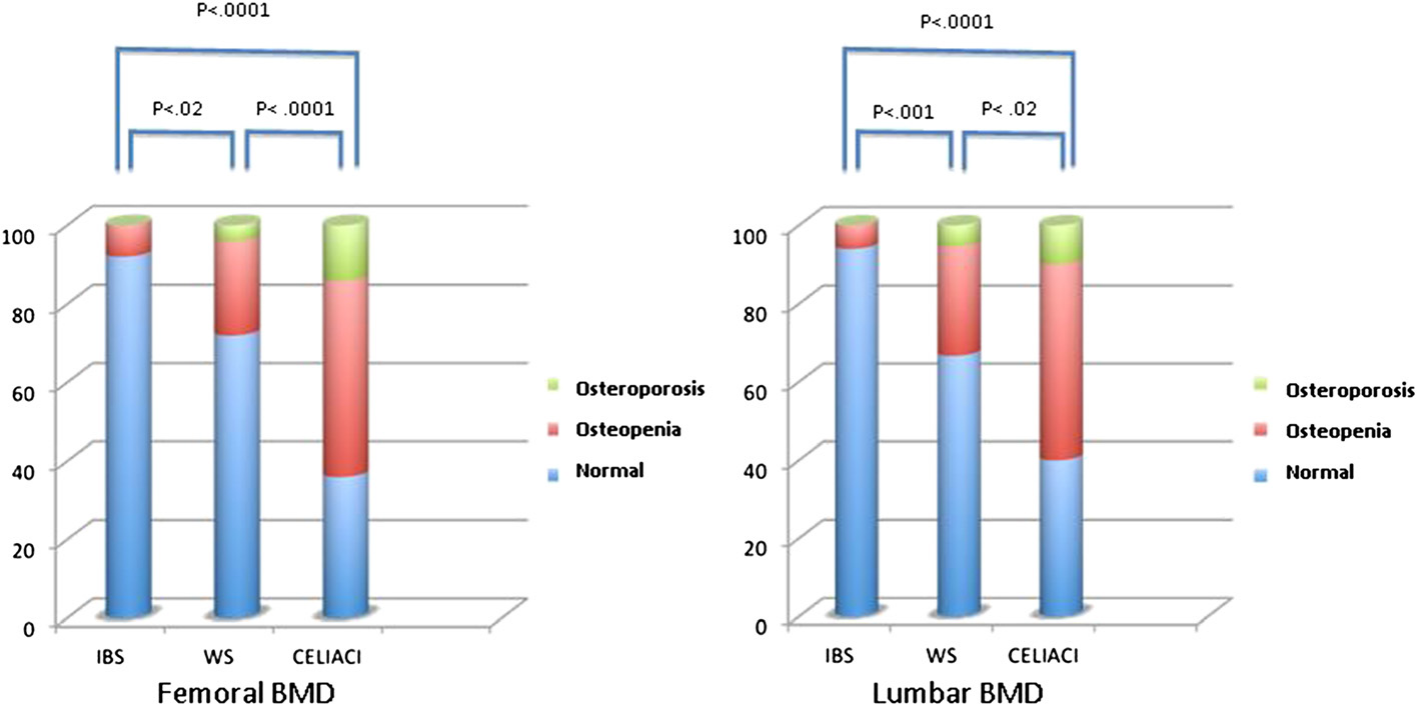
**Percentage of the patients of the three groups who had normal BMD, osteopenia or osteoporosis, evaluated both at the lumbar spine and femoral neck.** The frequency of osteopenia and osteoporosis was significantly different in the three groups, increasing from IBS to NCWS, and to CD patients (*P* <0.0001, Spearman’s rank correlation). BMD, bone mass density; CD, celiac disease; IBS, irritable bowel syndrome; NCWS, non-celiac wheat sensitivity.

Figure 2 shows the percentage of patients of the three groups who had normal BMD, osteopenia or osteoporosis, evaluated both at the femoral neck and at the lumbar spine. The frequency of osteopenia and osteoporosis increased from IBS to NCWS, and to CD patients (*P* <0.0001, Spearman’s rank correlation). Osteopenia or osteoporosis was found in 28% of the NCWS patients at the femoral neck and in 34% at the lumbar spine, whereas only 6% (lumbar spine) to 8% (femoral neck) of IBS patients had osteopenia and none of them had osteoporosis (for femoral neck *P* <0.02, chi-square 7.8; for lumbar spine *P* <0.001, chi-square 13). In total, 35 NCWS patients (46.6%) had osteopenia or osteoporosis at one of the two sites (femoral neck or lumbar spine). CD patients had a higher frequency of osteopenia (50%) and osteoporosis (14%) than both NCWS and IBS patients.

Table 2 shows the frequency of some clinical, histological and genetic findings in the NCWS patients with and without reduced BMD. In patients with NCWS and osteopenia or osteoporosis, in comparison to the patients with normal BMD, there was a significantly higher frequency of subjects with BMI values <20 (Mantel-Haenszel = 5.7, *P* <0.02), and with hemoglobin values <12 gr/dl (Mantel-Haenszel = 10.1, *P* <0.001), and of subjects with multiple food sensitivity (Mantel-Haenszel = 7.8, *P* <0.005). The presence of osteopenia and osteoporosis did not correlate with the duodenal histology findings (Marsh 0 or Marsh 1 and presence/absence of eosinophil infiltration), nor with the presence of the DQ2 or DQ8 alleles, or with the serum level of albumin and triglycerides (data not shown).

**Table 2:** **Distribution of some variables in NCWS patients with normal bone density, with osteopenia and with osteoporosis**

	**BMI <20**	**Hb <12**	**DQ2/DQ8**	**Marsh 1**	**Duodenal eosinophils**	**MFS**
Normal n = 40	19 (47%)	2 (5%)	22 (55%)	24 (60%)	15 (37%)	10 (25%)
Osteopenia n = 29	17 (59%)	7 (24%)	4 (14%)	12 (41%)	9 (31%)	16 (55%)
Osteoporosis n = 6	6 (100%)	3 (50%)	5 (83%)	3 (50%)	0 (0%)	4 (67%)

MI: χ2t = 5.7, *P* <0.02; Hb: χ2t = 10.1, *P* <0.001; multiple food sensitivity: χ2t = 7.8, *P* < 0.005; HLA DQ2/DQ8: χ2t = 1.2, *P* = ns; Marsh 1: χ2t = 1.4, *P* = ns; Duodenal eosinophils: χ2t = 2.5, *P* = ns. Duodenal lymphocytosis (Marsh 1) was defined as a number of CD3+ >25 IEL/100 epithelial cells. Duodenal eosinophils infiltration was defined as a number of eosinophils >60 per 10 high-power fields. BMI, body mass index; Hb, hemoglobin; IEL, intraepithelial lymphocytes; NCWS, non-celiac wheat sensitivity, MFS, multiple food sensitivity.

**Figure 3:**
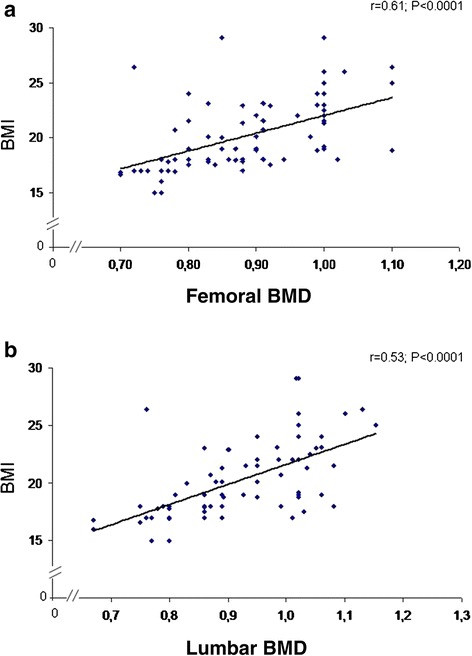
**Individual values of BMD, expressed as absolute values (g/cm** ^**2**^ **), in relation to the BMI, both at the femoral neck (Panel a) and at the lumbar spine (Panel b).** A significant direct correlation between the individual values of BMD (g/cm2) and the BMI values (*P* <0.0001) has been proved. BMD, bone mass density; BMI, body mass index.

Furthermore, in NCWS patients, there was a significant direct correlation between the individual values of BMD (g/cm2) and the BMI values (*P* <0.0001) (Figure 3). A similar, although less significant, correlation between BMD and BMI values was also found in IBS patients (r = 0.30, *P* <0.04) and in CD controls (r = 0.36, *P* <0.01).

Multiple regression analysis showed that the presence of osteopenia or osteoporosis was related exclusively to the BMI (*P* <0.002) and to multiple food sensitivity (*P* <0.03).

Mean values ± SD of daily dietary calcium intake in NCWS patients (considered as a group) were 615 ± 274 mg, significantly lower than in IBS controls (766 ± 284 mg; *P* <0.001) and in CD controls (821 ± 312 mg; *P* <0.001). There was a significant difference between patients with NCWS alone (calcium intake: median 770 mg/daily, range 300 to 1100 mg) and those with multiple food sensitivity (median 330 mg/daily, range 30 to 900 mg) (z = 28.8, *P* <0.0001, Mann–Whitney test).

Wheat exclusion from the diet before entering the study was self-reported in 21 of the 35 (60%) NCWS patients with osteopenia/osteoporosis and in 16 of the 40 (40%) patients without it (*P* <0.08, chi-square test). There was no difference in smoking habits or the levels of physical activity between the NCWS and IBS groups or within the NCWS group in the subjects with bone mass loss and those with normal bone mass.

## Discussion

An increasing number of papers have recently been published by many groups which seemed to confirm that ‘gluten-sensitivity’ should definitely be included in the spectrum of gluten-related disorders [2,3]. However, some doubts persist about the pathogenic mechanisms [9] and it has been suggested that wheat could cause gastrointestinal symptoms independently of its gluten-content [10-13]. For these reasons we suggested the term ‘non-celiac wheat-sensitivity’ [14].

A recent UK study showed that the self-reported prevalence of wheat-sensitivity was 13% [15]. The prevalence of strict adherence to a gluten-free diet in the general population ranged between 0.55 and 3.7% [15,16].

However, nothing is known about the osteoporotic risk in this condition, whereas it is known that more than 75% of untreated celiac patients suffer from a loss of bone mass [1].

We showed that the NCWS patients had a significantly lower bone mineral content than IBS controls both at the lumbar spine and at the femoral neck. Furthermore, 35 of the 75 NCWS patients (46.6%) had osteopenia or osteoporosis at one of the two sites. This high frequency of low bone mass in NCWS patients is much more relevant if it is considered that our study population included young subjects, mostly pre-menopausal women, with median age 36 years. These women will reach the age of menopause - the most critical period for bone loss risk - with bone mass already reduced.

Concerning the possible causes of osteopenia/osteoporosis in NCWS, we did not find any correlation with the degree of inflammation of the duodenal mucosa: there was no association with the Marsh 1 lesion (increased number of IELs) nor with the mucosal eosinophil infiltrate. The presence of the DQ2 or DQ8 HLA haplotypes was not associated with the presence of bone mass loss. On the whole these data seem to indicate that the mechanisms which cause osteoporosis in CD patients (malabsorption, inflammation, and so on) would not be pivotal in NCWS patients.

On the contrary, we found a direct correlation of bone mass loss with the BMI and hemoglobin values and these data could support the hypothesis of a role for malnutrition. In particular, it must be emphasized that about one third of our patient population reported weight loss of >10% of body weight during the last year, and that 42 NCWS patients (56%) had a BMI less than 20. Thus, the role of malnutrition seems very important in our study group.

We found that the dietary calcium intake of the NCWS patients (mean 615 mg/day) was significantly lower than that of the IBS controls, and much lower than the 1,000 mg/day commonly recommended. The selective malnutrition could be caused by self-restriction of the diet: 37 of the 75 NCWS patients reported self-exclusion of wheat before entering the study, and dietary calcium intake was significantly lower in patients with multiple food sensitivity than in patients with NCWS alone, probably because the latter patients excluded a higher number of foods from their diet. Bucci *et al*. reported 70% of NCWS patients excluded milk and dairy products from their diets to avoid symptoms [17]; Tavakkoli *et al*. reported that the people self-diagnosed as ‘wheat-sensitives’ had additional food avoidances including dairy (59%) [18]. A lower percentage (35%) of coexistent food intolerance has been recently reported in an Italian multicenter study [19]. This suggests that NCWS patients need dietary support to avoid mineral or other nutrient deficiencies.

However, the limits of our study must be highlighted. We did not suggest a low fermentable oligo-di-mono-saccharides and polyols (FODMAPs) diet to our patients, and it has been demonstrated that there was no evidence of effect of gluten in patients with gluten-sensitivity placed on diets low in FODMAP [13]. However, our patients became asymptomatic on the elimination diet and clearly reacted on DBPC wheat challenge, performed with a relatively small challenge dose of wheat. It is unlikely that our challenge method permitted us to identify many patients who are intolerant of the carbohydrate content of wheat, specifically fructans. Consequently, it is probable that we have studied a selected population which was different from that reported by Biesiekierski *et al*. [13]. Thus, our finding of the high prevalence of reduced bone mass and low BMI in NCGS patients must not be generalized.

We found a very high percentage of malnourished patients in our study group, but eating disorders were not regularly excluded by means of psychometric methods. In any case, the high percentage of NCWS patients with low BMI in our series could have influenced the high frequency of low BMD we found.

Furthermore, we cannot exclude that a percentage of our patients were in a ‘pre-celiac condition’, as could be suggested by the presence of DQ2/DQ8 haplotypes (41%), CD family history (24%) and duodenal lymphocytosis (52%). However, these are common findings in the NCWS population [2].

We were unable to provide data about Vitamin D levels, parathyroid hormone and markers of both bone synthesis and re-absorption; future studies need to clarify the possible mechanisms of osteoporosis in NCWS.

## Conclusions

We found an elevated frequency of bone mass loss in NCWS patients (46.6%). This was related to low BMI and was more frequent in patients with NCWS associated with other food sensitivity than in patients with NCWS alone. Dietary support should be strongly recommended at the time of NCWS diagnosis, whatever is its pathogenesis.

## Additional files

Additional file 1:
**Methods and diagnostic criteria in NCWS diagnosis.**
Additional file 2:
**Number of patients included/excluded during the different phases of the study.**
Additional file 3:
**VAS score comparison in patients suffering from wheat sensitivity alone (GROUP 1) and patients suffering from multiple food hypersensitivity (GROUP 2), under challenge with wheat, cow milk and placebo.**

## Abbreviations

AGA: anti-gliadin
anti-tTG: anti-transglutaminase
BMD: bone mass density
BMI: body mass index
CD: celiac disease
CMP: cow’s milk proteins
DBPC: double-blind placebo-controlled
DXA: dual energy x-ray absorptiometry
EmA: anti-endomysium
EOS: eosinophils
HPFs: high-power fields
IBS: irritable bowel syndrome
IELs: intraepithelial lymphocytes
MFS: multiple food sensitivity
NCGS: non-celiac gluten sensitivity
NCWS: non-celiac wheat sensitivity
VAS: visual analog scale
