# Supplementary material for: Risk of low bone mineral density and low body mass index in patients with non-celiac wheat-sensitivity: a prospective observation study
Source: BMC Med. 2014 Nov 28;12:230. doi: 10.1186/s12916-014-0230-2 (PMC4265355; doi:10.1186/s12916-014-0230-2)
Supplement: Additional file 1: — Methods and diagnostic criteria in NCWS diagnosis. [file 12916_2014_230_MOESM1_ESM.doc]

**NCWS diagnostic criteria**

The patients included met the following criteria: a) negative serum anti-transglutaminase (anti-tTG) and anti-endomysium (EmA) IgA and IgG antibodies; b) negative duodenal histology (absence of intestinal villous atrophy); c) negative IgE-mediated immune-allergy tests to wheat (skin prick tests and serum specific IgE detection).

**Laboratory methods**

On entering the study all patients underwent serum anti-tTG IgA and IgG, EmA IgA, and anti-gliadin (AGA) IgA and IgG assays performed using commercial kits (Eu-tTG IgA, anti-endomysium, and anti-gliadin IgA and IgG, Eurospital Pharma; Trieste, Italy), and reference values were determined as described previously (reference 11). Patients were also typed for HLA-DQ phenotypes by polymerase chain reaction using sequence-specific primers, with a rapid method (DQ-CD Typing Plus by BioDiaGene, Palermo, Italy) (reference 11). Specific IgE (RAST) and/or skin prick test for food allergens were performed on all patients, as previously described (reference 11).

**Bone mineral density assessment**

T-scores compared BMD with the mean of a healthy reference population, matched for sex and race, and were expressed as the number of standard deviations above or below the reference mean. Osteopenia was defined as at least one of the two DXA T-scores less than -1, whereas osteoporosis was diagnosed when either femoral neck or lumbar spine DXA T-scores were less than -2.5 (Report of a WHO Study Group Technical Report Series. Assessment of fracture risk and its application to screening for postmenopausal osteoporosis, No 843;1994.).

**Elimination diet and double-blind placebo-controlled (DBPC) challenge**

Food diaries were maintained by the patients during the elimination diet period to assess dietary intake and adherence to the diet. After four weeks on elimination diet, they underwent DBPC challenges. The challenges were performed with the reintroduction of a single food at a time. Patients were randomized to receive either the “active food” or the placebo, according to

a computer-generated order, determined by an observer not involved in the study.

In the case of wheat, the DBPCC was performed with capsules coded A or B containing wheat or xylose, respectively. Capsules A or B were given for 2 consecutive weeks and then after 1 week of washout the patients received the other capsules for another 2 weeks (cross-over design). Wheat challenge was performed administering a daily dose of 13g of flour, equal to about 20g of bread. A total of 12 capsules daily were given three times daily, away from meals.

DBPC for cow’s milk was performed by administering capsules coded as A or B containing milk proteins (casein from bovine milk, lactoalbumin, lactoglobulin - daily dose 6g, equal to about 200ml of cow’s milk) or xylose, respectively. A total of 6 capsules daily were given three times daily, away from meals.

The codes of the capsules were broken at the end of the study and the investigators did not know the content of the capsules during the study period. Challenges for other foods in patients with suspected multiple food hypersensitivity were performed in an open fashion.

The challenges were stopped when clinical reactions occurred (increase in VAS score >30) for at least two consecutive days (onset of abdominal discomfort or pain, associated with a change in stool frequency and/or appearance). The challenges were considered positive if the same symptoms which had been initially present reappeared after their disappearance on elimination diet.

**Dietary Assessment**

The patients were carefully instructed in compiling the printed list of the foods; each subject recorded the daily consumption of each food for 7 days (Monday-Sunday). The less common foods not appearing on the printed list were also noted, together with their quantities. Particular attention was paid to the calcium intake: the consumption of foods representing the major sources of daily calcium intake in the Italian diet, such as typical Italian aged cheese (i.e. Parmesan), ricotta cheese and yogurt was recorded. The dietary form was administered through an individual face to-face interview.
