# Supplementary figures and images for: Risk of low bone mineral density and low body mass index in patients with non-celiac wheat-sensitivity: a prospective observation study
Source: BMC Med. 2014 Nov 28;12:230. doi: 10.1186/s12916-014-0230-2 (PMC4265355; doi:10.1186/s12916-014-0230-2)

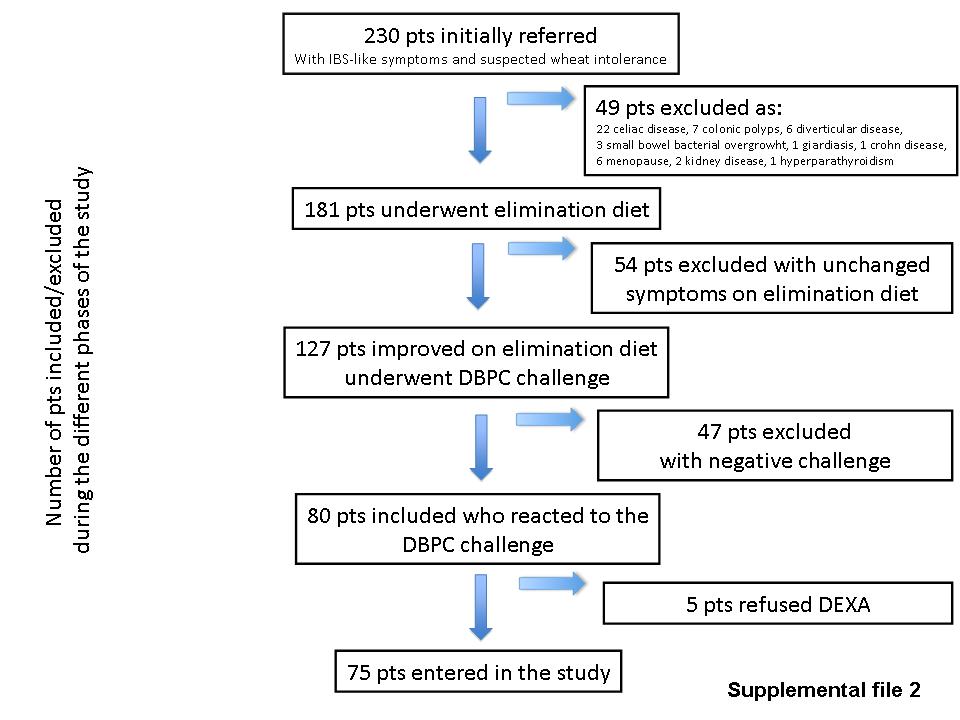

Supplement: Additional file 2: — Number of patients included/excluded during the different phases of the study. [file 12916_2014_230_MOESM2_ESM.jpeg]

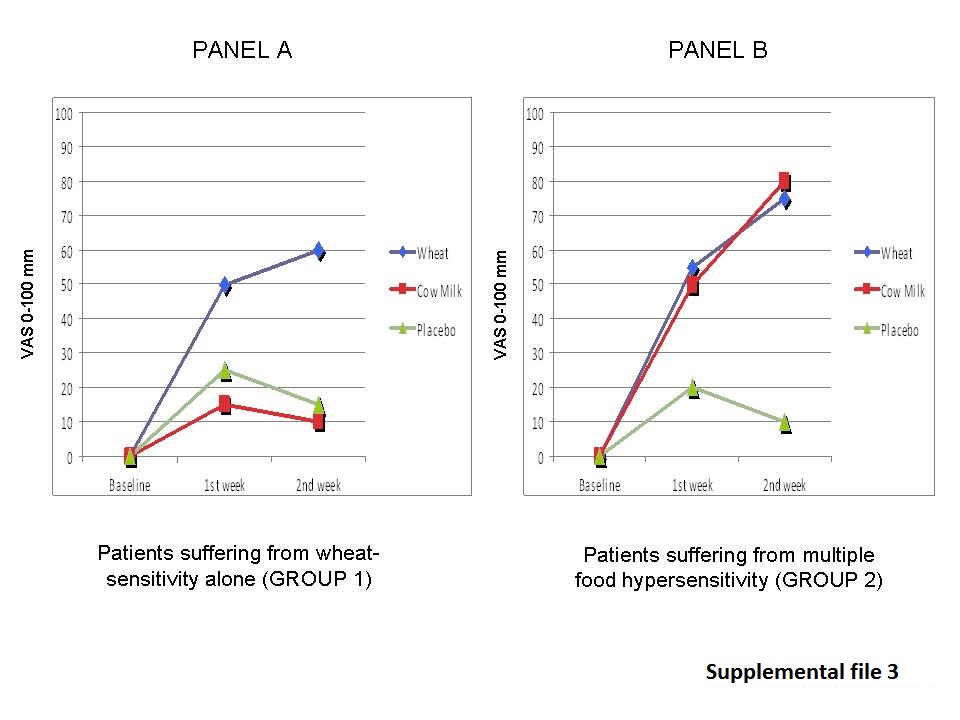

Supplement: Additional file 3: — VAS score comparison in patients suffering from wheat sensitivity alone (GROUP 1) and patients suffering from multiple food hypersensitivity (GROUP 2), under challenge with wheat, cow milk and placebo. [file 12916_2014_230_MOESM3_ESM.jpeg]
